# Change detection based on unsupervised sparse representation for fundus image pair

**DOI:** 10.1038/s41598-022-13754-5

**Published:** 2022-06-14

**Authors:** Yinghua Fu, Xing Zhao, Yong Liang, Tiejun Zhao, Chaoli Wang, Dawei Zhang

**Affiliations:** 1 School of Optical-Electrical and Computer Engineering, University of Shanghai for Science and Technology, Shanghai, China; 2 Collaborative Laboratory for Intelligent Science and Systems, Macau University of Science and Technology, Macau, China; 3 Department of Thoracic Surgery, Changhai Hospital of The Second Military Medical University, Shanghai, China; 4 Shanghai Institute of Intelligent Science and Technology, Tongji University, Shanghai, China

**Keywords:** Information technology, Experimental models of disease

## Abstract

Detecting changes is an important issue for ophthalmology to compare longitudinal fundus images at different stages and obtain change regions. Illumination variations bring distractions on the change regions by the pixel-by-pixel comparison. In this paper, a new unsupervised change detection method based on sparse representation classification (SRC) is proposed for the fundus image pair. First, the local neighborhood patches are extracted from the reference image to build a dictionary of the local background. Then the current image patch is represented sparsely and its background is reconstructed by the obtained dictionary. Finally, change regions are given through background subtracting. The SRC method can correct automatically illumination variations through the representation coefficients and filter local contrast and global intensity effectively. In experiments of this paper, the AUC and mAP values of SRC method are 0.9858 and 0.8647 respectively for the image pair with small lesions; the AUC and mAP values of the fusion method of IRHSF and SRC are 0.9892 and 0.9692 separately for the image pair with the big change region. Experiments show that the proposed method in this paper is more robust than RPCA for the illumination variations and can detect change regions more effectively than pixel-wised image differencing.

## Introduction

Detecting and predicating changes is one of the most commonly encountered low-level tasks in computer vision and video processing, the purpose of which is to identify the change regions between the two images of the same scene but taking at different times^1^. The goal is to identify the set of pixels significantly different between the current image and the previous reference image. Generally, the change regions should not contain unimportant changes such as differences caused camera motion, illumination variation and nonuniform attenuation. On one hand, as the change is of diversity, it is hard to know what kind of change will happen, hence the supervised learning techniques are often incapable in change detection. On the other hand, since the important and unimportant changes vary in different applications, it sometimes makes the algorithms predicate the changes difficultly^2,3^.

Given a pair of fundus images named reference image and current image separately, traditional unsupervised change detection methods consist of three steps: preprocessing, predicating a difference image and analyzing the difference image^4,5^. For the preprocessing step, the reference and current images are registered and adjusted in the intensity to each other. Then the image pair is compared pixel-pair-wisely to generate a difference image and the change regions are segmented from the difference image at last. Many techniques are used to analyze the difference image such as thresholding, classification or clustering. The change region heavily relies on the quality of the difference image^6^.

The preprocessing aims to filter unimportant changes and emphasize important changes of interest, which provides a sufficient preparation for producing a clear difference image. Important changes of interests mainly include the appearance or disappearance of objects, motion of objects relative to the background or shape changes of objects, changes of anatomic tissue structure in medical images^7,8^. Many preprocessing techniques are designed to reduce or remove unimportant changes such as registration and intensity normalization. Accumulated error and different imaging conditions make these technique challenging^9^. Choosing the proper preprocessing algorithms to stress important changes and depress unimportant changes is a significant step for image pair change detection.

The prediction of change regions mainly relies on pixel-by-pixel comparison such as image difference and image quotient in most applications^1,5,10^. Image difference is prevalent in most applications^1,5^. Image quotient is used in remote sensing field to reduce the speckle noise^10^. The pixel-by-pixel comparison is usually sensitive to illumination variations in the image and strongly dependent on the normalization of illumination. Background learning and modelling has attracted much attention^4,11–13^, which is widely used to detect video sequence anomalies from the beginning. The change regions are modelled as the foreground objects and obtained through subtracting the learned background from the current frames^14,15^. Principal component analysis (PCA) extracts the most of similar content linearly from a set of images^12,16^ or a serial^7,17^ as the background model and filters the noise or disturbance.

Robust PCA (RPCA) provides a good estimation for the background model which is robust to the global illumination variations^4,18^. After sufficiently sampling the learning frames, the illumination variations between two continuous frames become very small and are absorbed into the background model. RPCA-based change detection method for the fundus image pair extends the reference image into a background serial and then learn a robust background model from the extended frames^4^. Change regions are obtained by subtracting the current frame from the background model. The RPCA method combines the internal illumination correction with the illumination normalization between images to reduce the influence of illumination, expands the image pair into an image serial with the low-rank component, and then performs low-rank decomposition to obtain the change region^4^. This method is robust to global illumination, but sensitive to local illumination.

The change detection of image pairs is widely studied in the field of remote sensing^19–21^. A change detection method based on deep learning for synthetic aperture radar images is proposed^19^, which overlooks generating the difference image and produces the change probability map. Such technique is robust to the registration error and illumination variation, but training the network architecture needs a lot of labelled image pairs which is a huge challenge for medical image analysis^22^. Insufficient training samples hardly produce the accurate change regions. Further more the network architecture is retrained again when used in a different application domains.

In this paper, a change detection method based on unsupervised sparse representation classification (SRC) is proposed to produce a clear difference image. Generally, the original image pair without being preprocessed has complex illumination variations. The SRC technique models and reconstructs the background patches of the current image by sparse representation under a special dictionary composed of local neighborhood patches of the corresponding region in the reference imag, and the background of the current image is reconstructed under the given dictionary from the reference image. Change regions are obtained by background subtraction at the end.

The contributions of this paper can be summarized in following aspects. First, a change detection based on background modelling and subtraction is proposed where the background of the current image is reconstructed by the patch-based SRC and the local dictionary is learned from the corresponding region of the reference image. Second, the local illumination variations between two fundus images are decreased automatically during reconstructing the local background through adjusting the representation coefficients in the given dictionary. Third, for the big change regions, SRC can provide a coarse location, and it can combine with the other techniques to locate the change region with a high accuracy.

## Motivation

In most cases, it is difficult to collect a large amount of data from retinal fundus image pairs because some changes are rare and it is challenging to predict them. The change has never happened before, the supervised algorithm can not predict it accurately, and the unsupervised learning can be used to solve this kind of problems^23,24^.

Nonuniform illumination is common in the image pair and has a critical impact on the unsupervised change detection algorithms^1,25^. Illumination variations bring much distraction on predicating the change^26,27^. Many researchers put great efforts on designing various models to deal with the illumination variations^28,29^. The iterative robust homomorphic surface fitting (IRHSF) is specially conceived to model the illumination for the fundus images by calculating the curvature of the retinal surface^5^.

**Figure 1:**
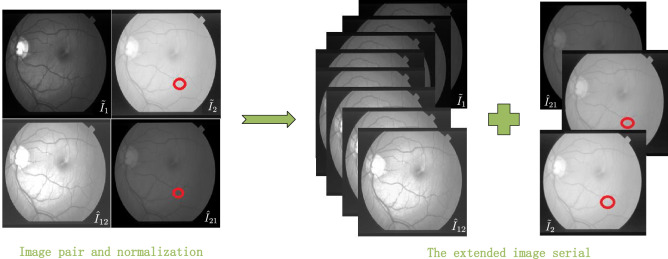
Serial expansion by linear interpolation.

For the fundus image pair with the reference image $I_{1}$ and current image $I_{2}$, RPCA-based change detection takes the main content of $I_{1}$ as the background model of $I_{2}$^4^. The goal of change detection is to estimate the background of $I_{2}$ from the reference image $I_{1}$, and make the illumination of the estimated background close to that of $I_{2}$, so as to reduce the interference of illumination variations and generate a clear difference image by subtracting the estimated background from $I_{2}$. Fu et al.^4^ combines the inter-image illumination with intensity normalization and the intra-image intensity correction together to reduce the intensity distractions and obtain a robust background model, as Fig. 1 shows. The first row is the image pair by the intra-image correction technique and the second is the normalized image in the left side of Fig. 1 and the red circle marks the attached noise patch.

$I_1$ and $I_2$ are adjusted first to $\tilde{I}_1$ and $\tilde{I}_2$ by the intra-image correction technique, then their intensity is normalized to each other according to the following formula:

1 $$\begin{aligned} \hat{I}_{12}=\frac{{\sigma _2}}{{\sigma _1}}\{\tilde{I}_1-\mu _1\}+\mu _2 \end{aligned}$$

and

2 $$\begin{aligned} \hat{I}_{21}=\frac{{\sigma _1}}{{\sigma _2}}\{\tilde{I}_2 -\mu _2\}+\mu _1 \end{aligned}$$

where $\mu _i$ and $\sigma _i$ are the mean and standard deviation of the intensity value of $\tilde{I}_i$($i=1,2$) separately. So the image pair is adjusted into the pairs at two different intensity levels $\hat{I}_{12}$ and $\hat{I}_{21}$ as the second row of the left image in Fig. 1 shows. The columns indicate the reference image and the current image separately. The linear interpolation in each column, e.g. $\tilde{I}_1$ and $\hat{I}_{12}$ as well as $\tilde{I}_2$ and $\hat{I}_{21}$, is conducted to decrease the illumination variations further to obtain a robust background model.

**Figure 2:**
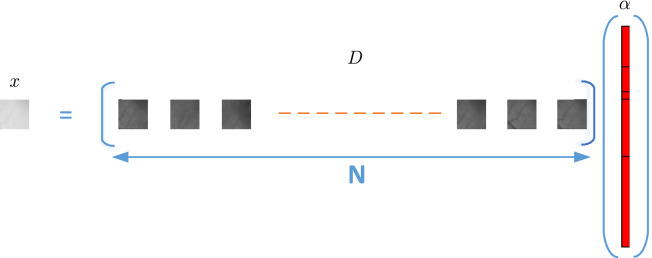
The local reconstruction and dictionary sparse representation of current image patch.

Although RPCA-based change detection takes a great effort to deal with the illumination variations and obtain a robust background model between the image pairs, it can’t remove the local illumination distraction. In order to get rid of the local illumination, the local background of $I_2$ is modelled by SRC in this paper. The background of the current fundus image is estimated and reconstructed patch-by-patch by the dictionary and sparse representation with a sliding window as Fig. 2 shows.

The background of the local region patch in $I_2$ is estimated by the neighborhood patches in $I_1$. Aside from the content of the patch, illumination variations between the reference patch and current patch can be corrected by the representation coefficients under the neighborhood dictionary in $I_1$. The main information in $I_1$ is encoded and transferred through dictionary mapping, as well as the intensity of the image is automatically adjusted by sparse representation coefficient. By doing this, it avoids effectively the distraction of the complex illumination on the change regions.

In addition, sparse representation can automatically denoise and is robust to the interference caused by the small camera movement and registration error. The SRC-based change detection method takes into account the local neighborhood information more effectively than the comparison on point pairs and the global RPCA. Hence it detects the change region more effectively and generates cleaner and clearer change regions.

## Results

**Figure 3:**
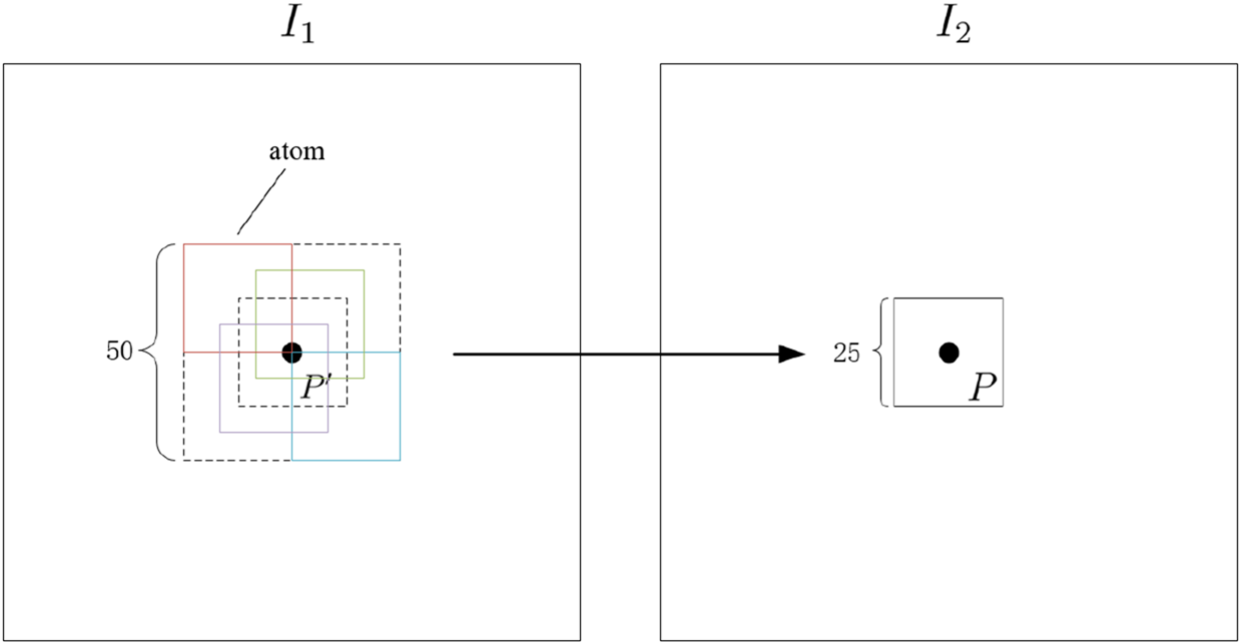
The construction of local dictionary.

The SRC-based change detection algorithm proposed in this paper is verified by clinical data of retinal fundus images. The retinal image pair is registered by the partial intensity invariant feature descriptor (PIIFD) algorithm^30^. As shown in Fig. 3, the local dictionary is composed of 25 neighborhood patches of size $25\times 25$, which are distributed in a square region with a side length of 50 pixels and the center is located on the pixel $P'$ in the reference image, which corresponds to the pixel *P* of the current image. In this paper, let $\lambda = \frac{1}{m}$, where *m* is the total number of pixels of the image.

### Clinical data

This paper verifies the proposed algorithm from three pairs of clinical data: a pair of images containing small lesions, a pair of images containing complex lesions and a pair of images containing large lesions.

**Figure 4:**
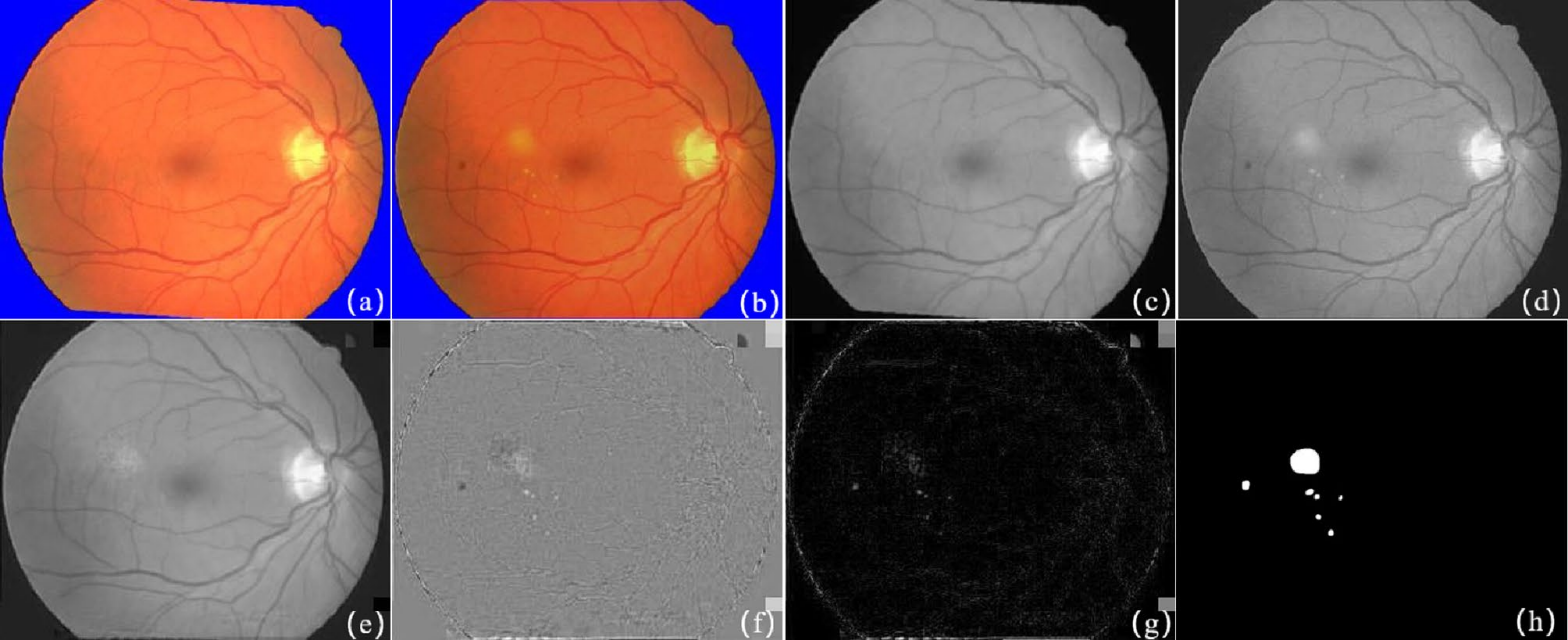
The result of image pair with small lesions. (**a**) Reference image; (**b**) current image; (**c**) grayscale image of (**a**); (**d**) grayscale image of (**b**); (**e**) the reconstructed background of (**d**) based on the proposed method; (**f**) the difference image of (**d**) and (**e**); (**g**) the absolute difference image of (**d**) and (**e**); (**h**) the ground-truth.

The first pair of raw images shown in Fig. 4a,b are from the literature^31^, and the resolution of the image is $338 \times 276$. Figure 4a is a normal retinal fundus image, and Fig. 4b contains several small lesions. Figure 4c,d are the grayscale images of Fig. 4a,b separately. Figure 4f gives the result of background subtraction of Fig. 4d,e,g shows the detected change region by a absolute difference image. As Fig. 4f,g shows, the bright dots indicates the small lesions not existing in the reference image but significantly different from noise. Figure 4h illustrates the groundtruth of the change regions. This experiment shows SRC change detection method has a good performance on detecting small lesions.

**Figure 5:**
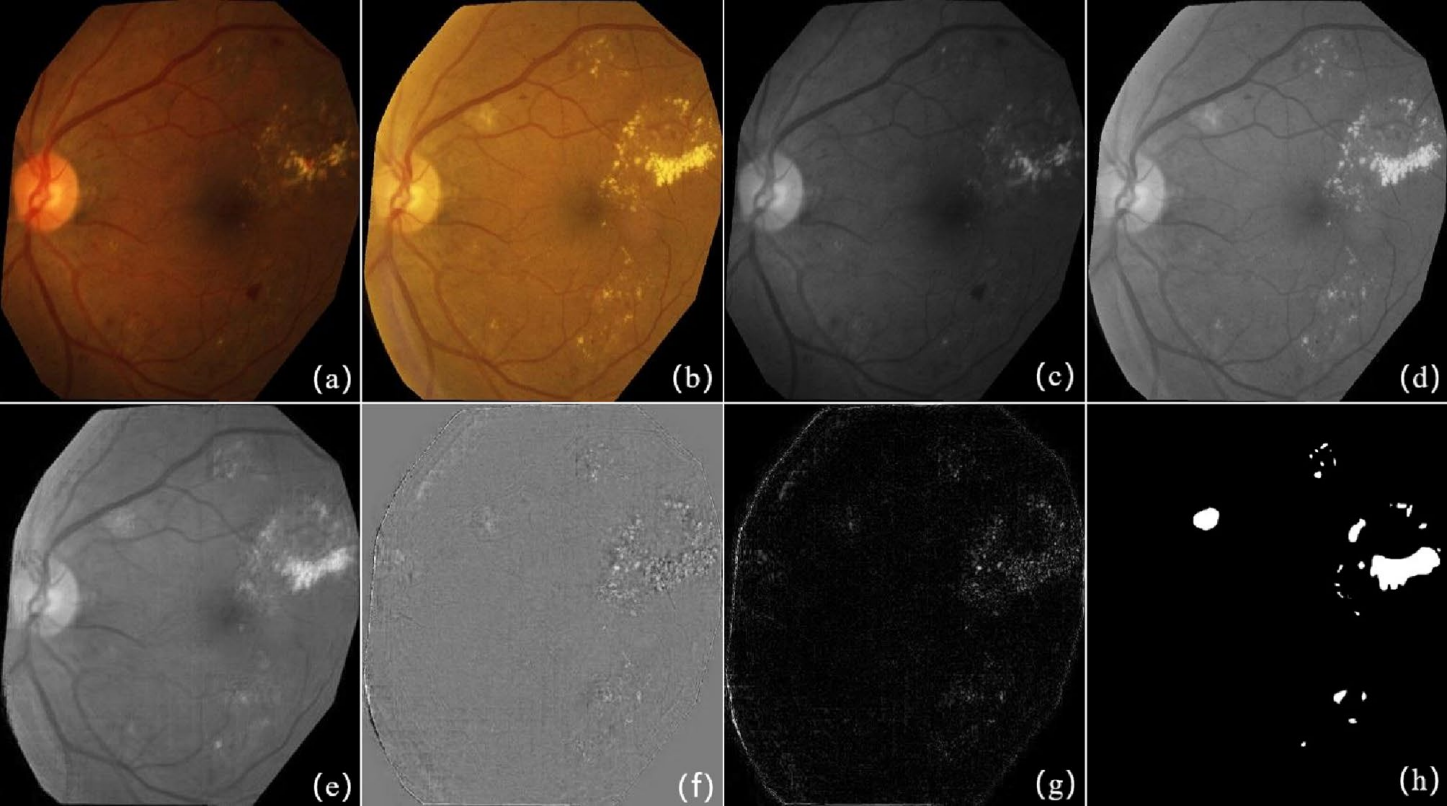
The result of image pair with complex lesions. (**a**) Reference image; (**b**) current image; (**c**) grayscale image of (**a**); (**d**) grayscale image of (**b**); (**e**) the reconstructed background of (**d**) based on the proposed method; (**f**) the difference image of (**d**) and (**e**); (**g**) the absolute difference image of (**d**) and (**e**); (**h**) the ground-truth.

The second pair including complex illumination variations shown in Fig. 5a,b comes from the literature^5^, and the experimental results are shown in Fig. 5e–g. Figure 5a has the dark global intensity and uneven local illumination variations in the right side and Fig. 5b has the bright global intensity. The gray images of Fig. 5a,b are shown in Fig. 5c,d. The reconstructed background of Fig. 5d and the absolute difference image are presents in Fig. 5e,g separately. Figure 5h illustrates the groundtruth of the change regions. Although there are great illuminations between this image pair, the reconstructed background has great similarity with Fig. 5d and there is less distraction of illumination on the difference image.

**Figure 6:**
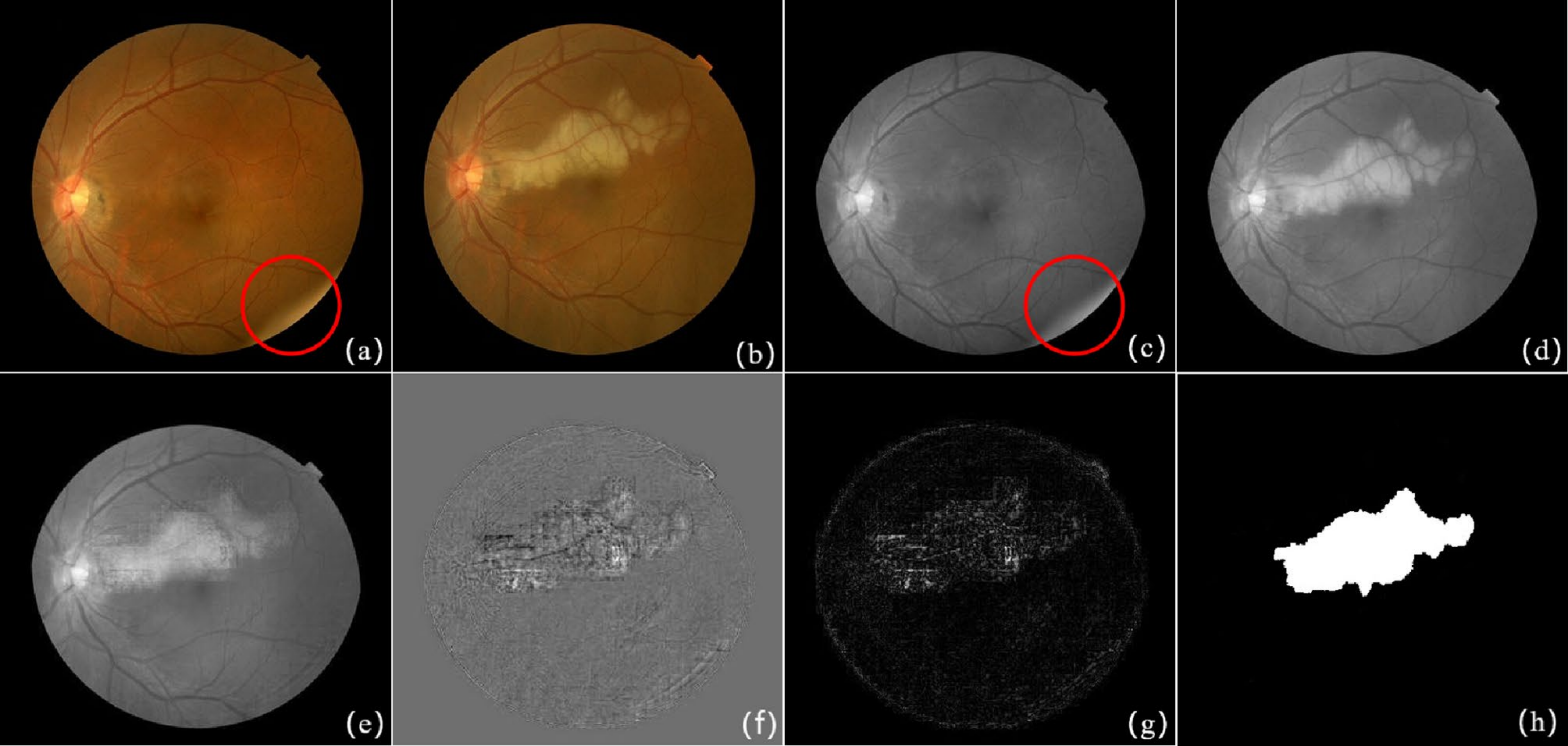
The result of image pair with large lesions. (**a**) Reference image; (**b**) current image; (**c**) grayscale image of (**a**); (**d**) grayscale image of (**b**); (**e**) the reconstructed background of (**d**) based on the proposed method; (**f**) the difference image of (**d**) and (**e**); (**g**) the absolute difference image of (**d**) and (**e**); (**h**) the ground-truth.

The third pair was collected from the ophthalmology clinic of Shanghai Xinhua Hospital, as shown in Fig. 6a,b. The current image Fig. 6b contains a large lesion region, and the red circle in Fig. 6a marks the randomly illumination. Figure 6c,d are the grayscale image of Fig. 6a,b separately. Figure 6e is the reconstructed background image, and random illumination in the reference image is not used as a background expression. Figure 6f gives the difference image of Fig. 6d,e. Figure 6g shows the detected change region through absolute difference operation, and Fig. 6h presents the groundtruth of the big change region. The detection result shows that patch-based SRC method adaptively corrects local and global intensity and locate highlight large lesions and is robust to illumination distraction.

### Combination of SRC and IRHSF

The above experiments show that the SRC change detection method has a good detection effect for images with small change regions, but the detection results are not ideal for the cases involving the large change regions. When the lesions of the current image are large, the background reconstructed by SRC method still involves much lesion information, it is difficult to detect large, connected change regions, as the large bright lesion region shown in Fig. 6g. Since the interior zone of the large lesion can be represented by subordinate component in the dictionary as a normal structure, the background can’t be reconstructed right which leads to the change region in Fig. 6e blurry. However SRC is robust to illumination and can be used as a coarse location of the big change regions, and it combines with IRHSF illumination correction together to improve the change detection results for the retinal fundus image pair.

**Figure 7:**
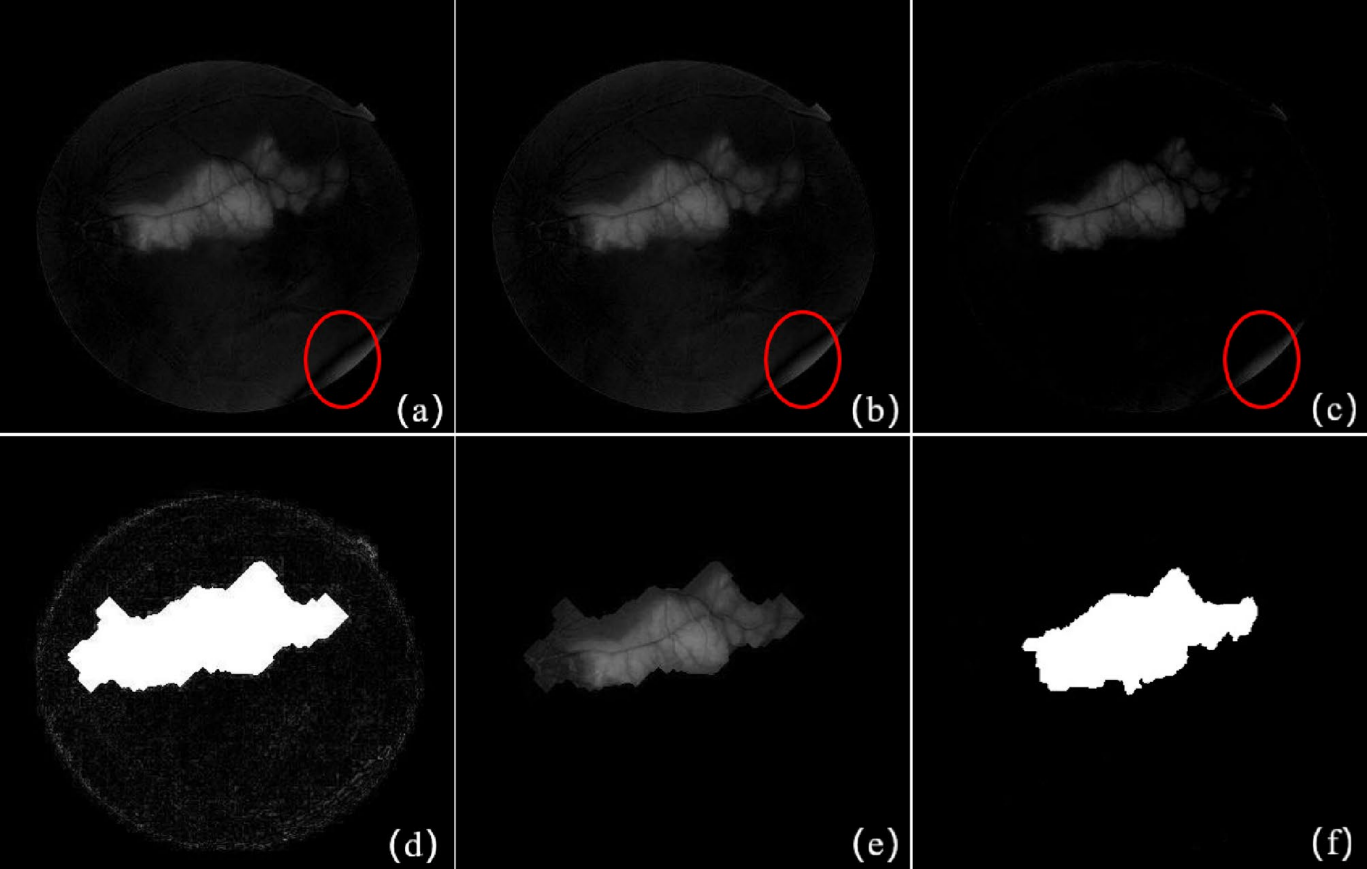
The result of the pair of images with large lesions in Fig. 6. (**a**) Result of global difference method; (**b**) difference image with IRHSF correction; (**c**) result of RPCA method; (**d**) change area mask of interest; (**e**) result fusing IRHSF and SRC; (**f**) the ground-truth.

IRHSF is a normalized model proposed by Narasimha-Iyer special designed for illumination correction of digital retinal fundus images^5^. This method has a good effect on correcting the intra-image intensity in the fundus image. However it needs to locate and segment the anatomical structure accurately first before calculating of the illumination model, which is a great load for modelling. The differential operation based on IRHSF illumination correction is one of the main methods for detecting changes in retinal images^5^. Combining SRC and IRHSF can remove the random illumination distraction and detect more change regions, as Fig. 7 illustrates.

The SRC detection result in Fig. 6g is binarized and morphologically operated to generate a change region mask as shows in Fig. 7d, and then superimposed on the IRHSF difference image to filter out the illumination. Figure 7a is the global difference detection result after the registration, Fig. 7b is the difference image based on IRHSF correction, and Fig. 7c is the result of the RPCA change detection^32^. As the above-mentioned analysis, the results of all the three techniques are distracted by the random illumination marked by the red circle. Figure 7e gives the change region detected by the combination of SRC and IRHSF correction. The detection results fusing SRC and IRHSF are cleaner and remove the local illumination.

**Figure 8:**
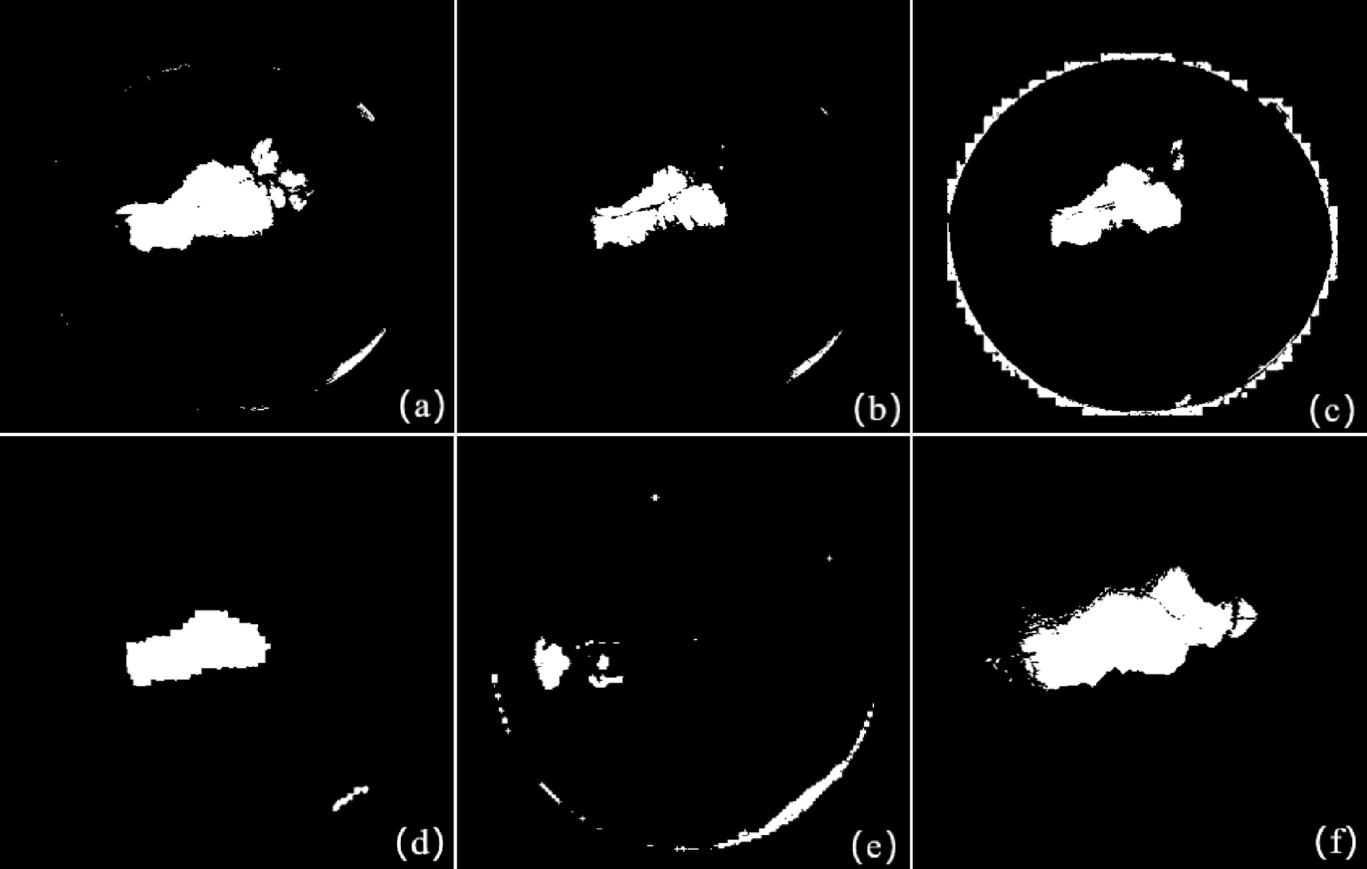
Binary CMs of the pair of images with large lesions in Fig. 6. (**a**) Binary CM of IRHSF; (**b**) binary CM of RPCA; (**c**) binary CM of NRELM; (**d**) binary CM of IRG-McS; (**e**) binary CM of NPSG; (**f**) binary CM of the fusion method of IRHSF and SRC.

In order to further evaluate the binary change map (CM) based on the fusion method of IRHSF and SRC for the image pair with large lesions, five methods are used for comparison: IRHSF, RPCA, NRELM^33^, IRG-McS^34^ and NPSG^20^. Among them, IRHSF, RPCA, NPSG and the fusion method of IRHSF and SRC generate difference images, we adopt the thresholding method to generate binary CMs, the threshold is uniformly set to 0.15. For the NRELM and IRG-McS, they directly generate binary CMs. It should be noted that NRELM, IRG-McS and NPSG use the codes provided in their papers, and the parameter settings are consistent with the papers. Figure 8 shows binary CMs generated by these six methods respectively, and the ground-truth is shown in Fig. 7f.

## Discussion

The prevalent change detection method for the image pair is image differencing, and the difference image is usually generated by subtraction or ratio operator^1,5^. Image differencing based on intensity subtraction is sensitive to illumination variations and calibration errors^5,10^. Image ratioing compares the image pair pixel-to-pixel on ratio^35–37^, which is designed to deal with the speckle noise for the synthetic aperture radar (SAR) images. It is robust to noise but fails on detecting small lesions.

Gong et al.^35^ combine a mean-ratio operation with a log-ratio operation to generate the difference image. Such a fusion method takes the dominant advantage in big lesions but the small lesions are filtered as noises. The SRC change detection method is more robust than the traditional differential method to the uneven illumination of the image, and achieves a good performance in the detection of small lesion changes. For large lesion changes, the SRC method is combined with other change detection methods to coarsely locate the change region, making the detected change region cleaner and clearer.

This section gives the ROC curve and PR curve of the experimental quantitative analysis of the aforementioned change detection methods. AUC (the area under ROC curve) designed to evaluate the comprehensive performance of the classifier is calculated through ROC curve where TPR and FPR denote the vertical and horizaontal coordinates separately. MAP (the area below PR curve) is used to calculate the average accuracy value through PR curve where Precision and Recall denote the vertical and horizaontal coordinates respectively. The four indexes are given by the following formulas:

3 $$\begin{aligned} TPR=\frac{TP}{TP+FN} \end{aligned}$$

4 $$\begin{aligned} FPR=\frac{FP}{TN+FP} \end{aligned}$$

5 $$\begin{aligned} Precision=\frac{TP}{TP+FP} \end{aligned}$$

6 $$\begin{aligned} Recall=\frac{TP}{TP+FN} \end{aligned}$$

where TP, FP, TN and FN indicate true positive, false positive, true negative and false negative, respectively. The four indexes are calculated through statistical analysis of each pixel in the image. Among them, true positive means detecting the correct positive sample, false positive means the false detection is true negative sample, and so on.

**Figure 9:**
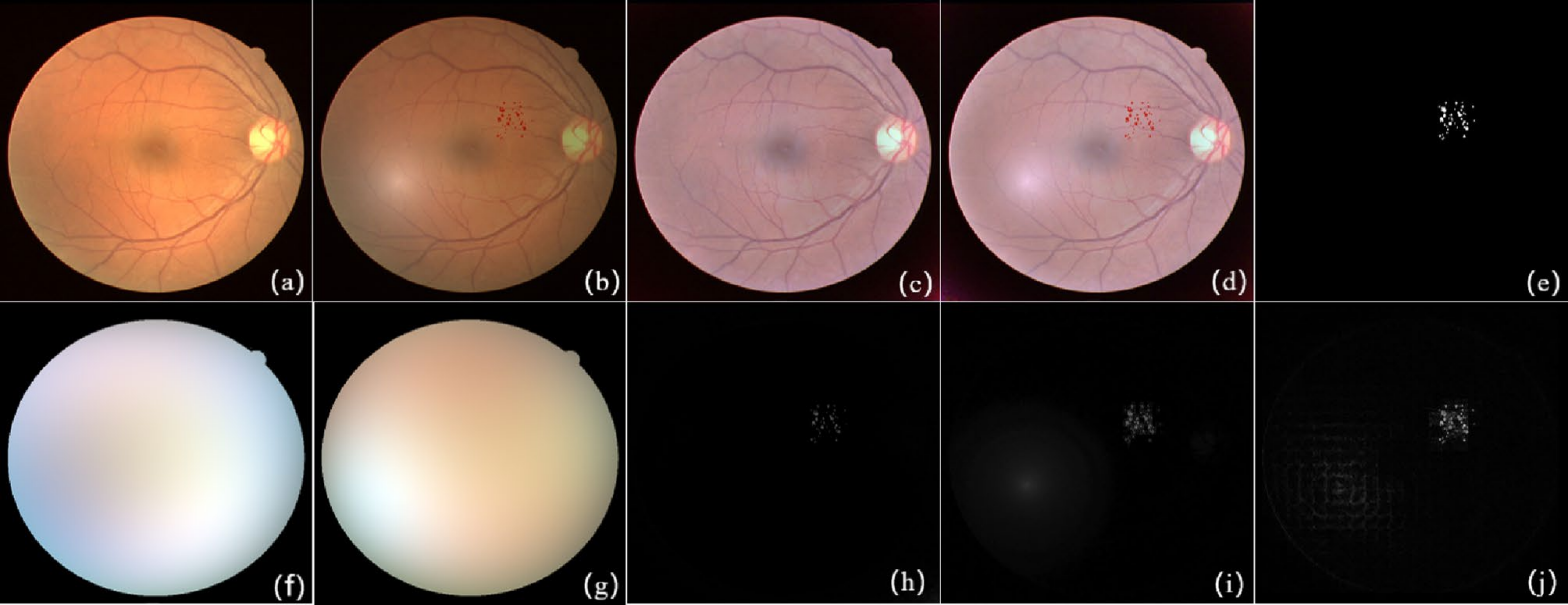
Simulated small lesion image pairs results. (**a**) Reference image; (**b**) current image; (**c**) the corrected reference image with IRHSF; (**d**) the corrected current image with IRHSF; (**e**) the ground-truth; (**f**) the illumination model of the reference image; (**g**) the illumination model of the current image; (**h**) detection result of IRHSF; (**i**) detection result of RPCA; (**j**) detection result of the proposed method in this paper.

Figure 9 shows the IRHSF, RPCA and SRC detection results of small lesion image pairs. The reference image Fig. 9a from a normal image of DRIVE dataset, and the current image Fig. 9b is pasted small noise spots, and its local brightness is adjusted. However before the RPCA and SRC methods, there is no any illumination correction except graying the color image pair of Fig. 9a,b.

The detection results of RPCA showed obvious bright spot distraction and the detected small lesions were not clear in brightness. The results displayed by SRC were less distracted by the light spots than the RPCA method, and the change regions of the small lesions were clear and cleaner. This experiment shows that the SRC method is more robust to local illumination variations than RPCA. The complexity of IRHSF is at least $M^{2}$ order^5^. However the complexity of RPCA and SRC is *M* order. Therefore, IRHSF illumination correction is usually time-consuming and the algorithm is not as efficient as RPCA and SRC.

**Figure 10:**
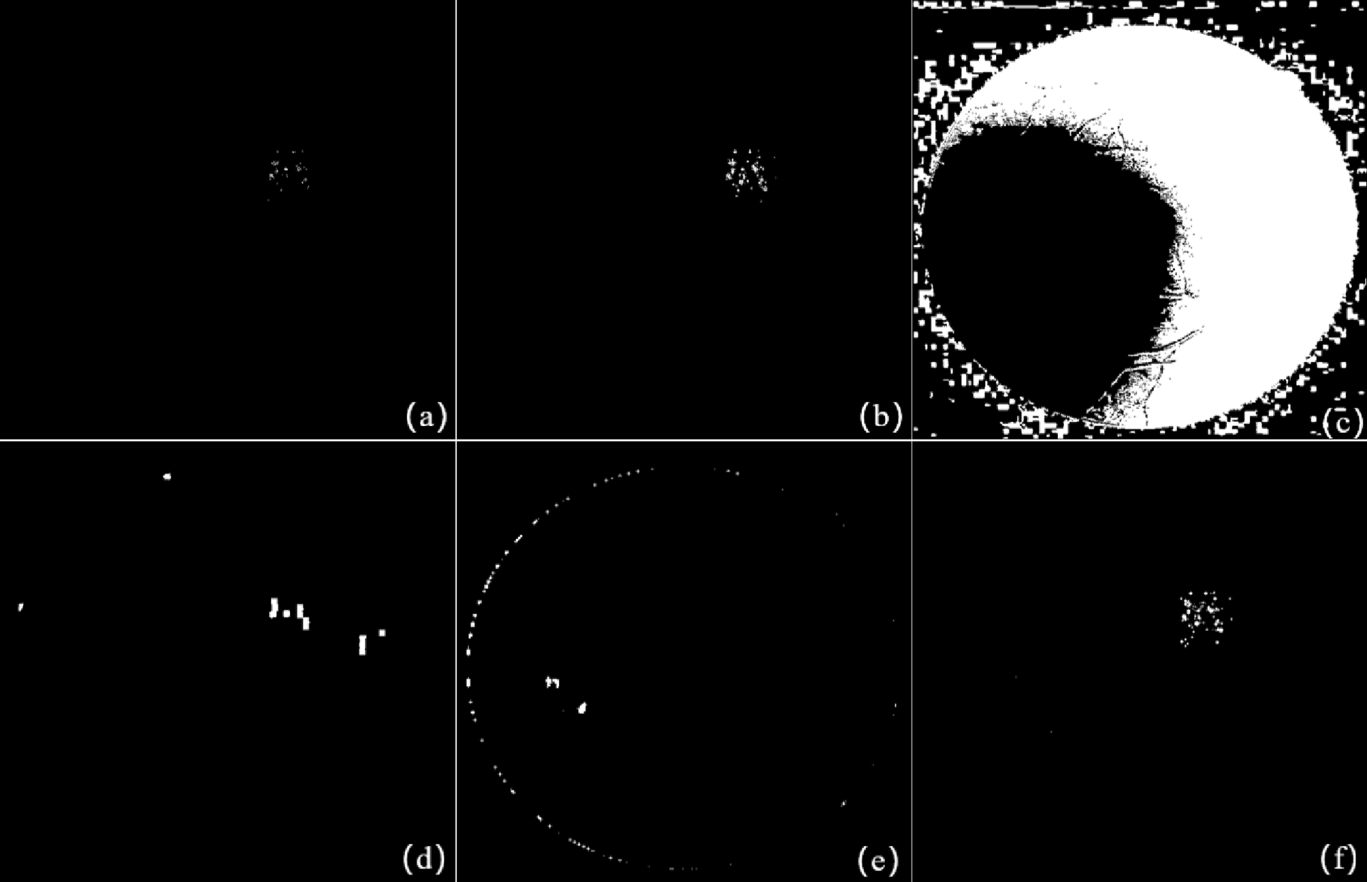
Binary CMs of the pair of images with small lesions in Fig. 9. (**a**) Binary CM of IRHSF; (**b**) binary CM of RPCA; (**c**) binary CM of NRELM; (**d**) binary CM of IRG-McS; (**e**) binary CM of NPSG; (**f**) binary CM of SRC.

Six methods: IRHSF, RPCA, NRELM^33^, IRG-McS^34^, NPSG^20^ and SRC are used to evaluate binary CMs for the image pair with small lesions in Fig. 9. For the difference images generated by IRHSF, RPCA, NPSG and SRC, a threshold value of 0.3 is uniformly set to generate binary CMs. NRELM and IRG-McS directly generate binary CMs. Figure 10 shows binary CMs generated by these six methods respectively, and the ground-truth is shown in Fig. 9e.

**Figure 11:**
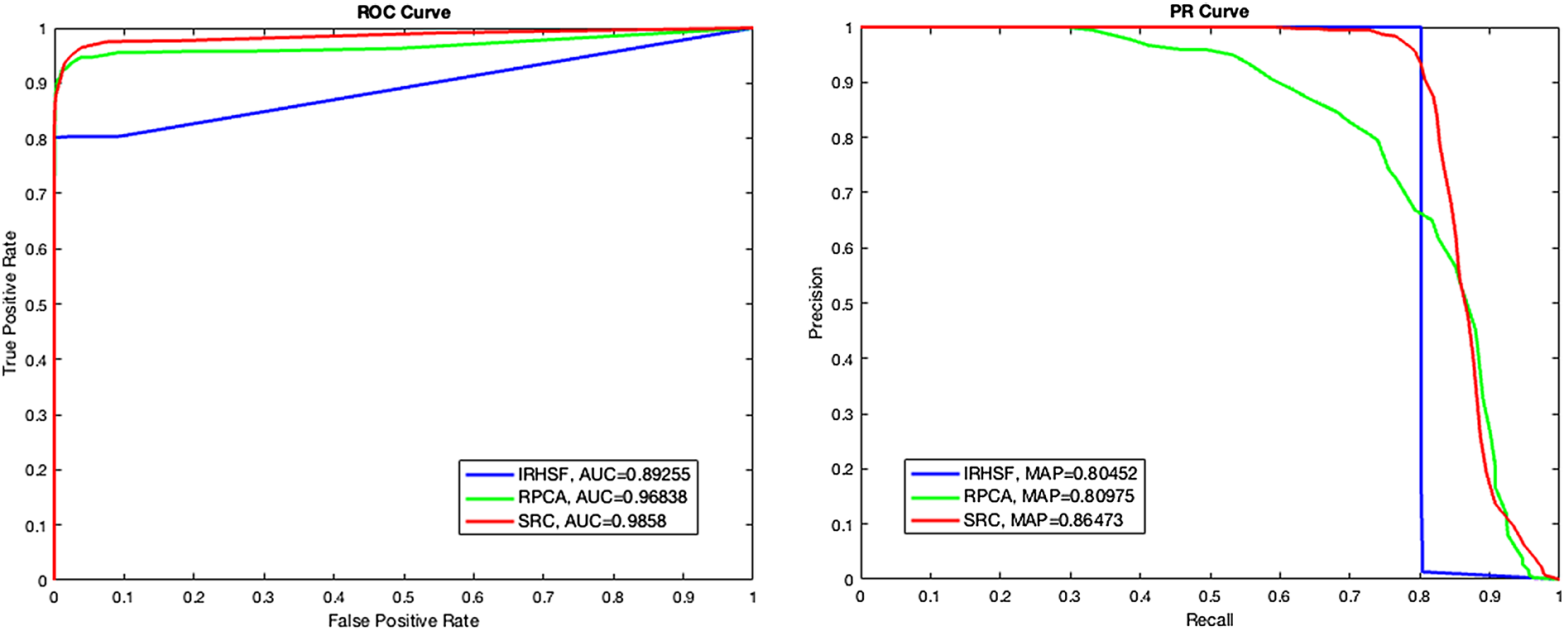
ROC and PR curves of three discussed methods based on the image pair with small lesions.

**Figure 12:**
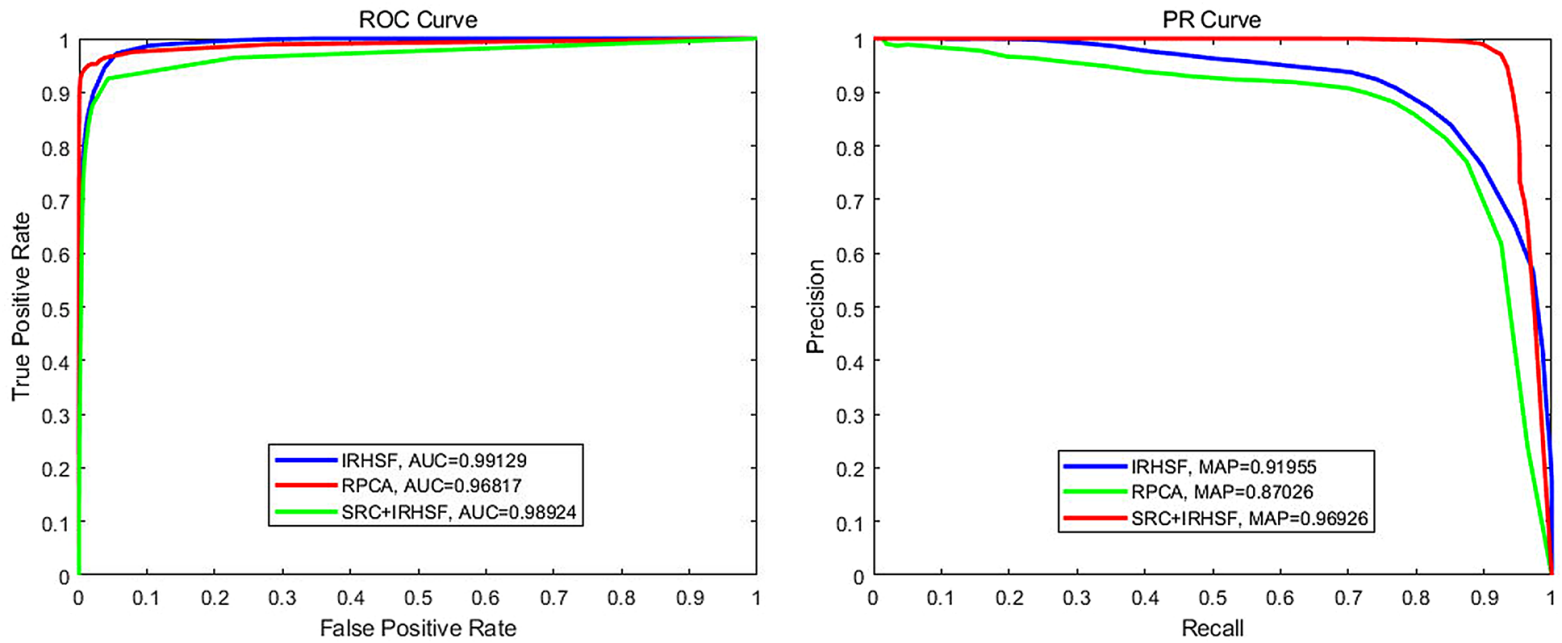
ROC and PR curves of three discussed methods based on the image pair with the big change region.

The ROC curves and PR curves of the IRHSF, RPCA and SRC are shown in Fig. 11. The AUC and mAP values of the SRC method with small lesions are 0.9858 and 0.8647, respectively. Figure 12 shows the ROC and PR curves of the IRHSF, RPCA and the fusion method of IRHSF and SRC for the image pair with big change region shown in Fig. 6a,b. The blue line indicates the IRHSF method, the green line indicates the RPCA method, and the red line is for the result of the combination of the SRC and IRHSF methods. The AUC of these three methods are very close, which are 0.99129, 0.96817 and 0.98925 respectively; their mAPs are 0.91955, 0.87026 and 0.96926 separately. The fusion method of SRC and IRHSF was superior to the the other two methods, and the change region generated by the fusion method was more accurate and cleaner than that of the other two methods.

Table 1 shows Intersection over Union (IOU) of binary CMs for the image pair with small lesions shown in Fig. 9a,b and big change region shown in Fig. 6a,b. Table 1 shows SRC obtains the highest IOU with small lesions, which is 0.7227. After combining with IRHSF, SRC gets the highest IOU with 0.855, which improves the original value of IRHSF 0.6179 over 0.235. Star means the fusion result of SRC and IRHSF in Table 1. These results show that the binary CM generated by the proposed method are better than those CMs generated by other methods.

**Table 1:** Intersection over Union (IOU) of binary CMs for the image pair.

Methods	IRHSF	RPCA	NRELM	IRG-McS	NPSG	SRC
Small lesions	0.1593	0.555	0.0035	0.1767	0	0.7227
Big change region	0.6179	0.3486	0.2702	0.4355	0.0783	0.855*

## Conclusion

This paper presents a change detection method based on SRC for the fundus image pairs. The change detection method proposed in this paper considers the more local neighborhood information compared with the methods based on point-by-point pairs and global RPCA. It can effectively reduce the distractions of illumination and is robust to the interference caused by small camera movement and registration error. The main advantages of this method are as follows: firstly, the SRC change detection method can reconstruct the background of the current image based on the local region information of the reference image, and then obtain the change region by background subtraction; secondly, the SRC method is robust to the distraction of illumination, and the local and global illumination variations between image pairs are automatically adjusted by sparse representation, hence the change mask is less affected by illumination; Finally, SRC is combined with the traditional change detection methods such as IRHSF to detect the change region more effectively.

As the size of atoms and the dictionary is important to the change detection and the reconstruction of the background, how to choose appropriate atoms to learn a good dictionary which can detect change regions with any size is the next work of this paper.

## Methodology

Sparse representation has achieved huge success and big breakthrough in many applications such as face recognition^32^, action recognition^38^, image processing^39^ and video background modelling^8^. For the image pair change detection, sparse representation can adaptively select the optimal image patches from the dictionary which is maximum linearly dependent with the reference image $I_1$.

### Sparse representation modelling

Given a dictionary *D* consisting of *N* *p*-dimensional atoms, *D* is a matrix with the size of $p\times N$, and a signal $x\in R^{p}$ is a *p*-dimensional vector. The procedure of sparse representation is to adaptively select some atoms that can best linearly represent the input signal *x* in order to let the reconstruction error between the recovered signal and the input signal minimized, which can be formulated as follows:

7 $$\begin{aligned} {\alpha ^*} = \arg \mathop {\min }\limits _\alpha \left\| {x - D\alpha } \right\| _2^2 + \lambda {\left\| \alpha \right\| _1} \end{aligned}$$

where $\alpha ^{*}$ is called sparse representation coefficients or sparse encoding of signal *x* under the dictionary *D*. ${\left\| \cdot \right\| }_{1}$ denotes the $l_{1}$ norm, which is used to determine the sparsity of the representation coefficient $\alpha$^40,41^. ${\left\| \cdot \right\| }_{2}$ denotes the $l_2$ norm, and $\left\| x-D \alpha \right\| _{2}^{2}$ is the square of the reconstructed error between the recovered signal $\hat{x}=D\alpha$ and the original signal *x*. $\lambda$ is a hyper-parameter to trade off the equilibrium between the sparsity and the reconstructed error. In fact, the larger $\lambda$ is, the more sparse $\alpha$ is and the less the non-zero elements of $\alpha$ are.

For SRC-based change detection proposed in this paper, the patch in $I_2$ is regarded as the vectorized input signal $x \in R^p$. The dictionary *D* is made of the corresponding neighborhood patches in $I_1$ as Fig. 2 shows. Generally, the covered region of the dictionary *D* in $I_1$ is larger than the current patch *x*. SRC-based change detection decompose the comparison of the whole scene to the two local neighborhoods in the fundus pair. When the change region is small, *x* has great similarity with the atoms in *D*. Hence the background of *x* excluding the change region can be represented by *D*, and the change region is regarded as the reconstruction error.

Current patch *x* is projected into the dictionary space *D* from the neighbor patches in $I_1$ and the background of *x* is linearly reconstructed by the sparse encoding coefficient $\alpha$ as the red block shows in Fig. 2. When *x* slides throughout $I_{2}$, the background of $I_{2}$ denoting $B_{2}$ is padded by the recovered background patches from *x*, which are reconstructed by the sparse representation of *D* in $I_{1}$.

For the *i*-th current patch $x_i$, $y_i$ denotes the *i*-th referent patch and ${{X}_{i}}=\{{{x}_{i1}},{{x}_{i2}},\ldots , {{x}_{iq}}\}$ denotes the *q* extracted neighborhood patches around $y_i$ in $I_{1}$ and $x_{ij}$ means the *j*-th patch for $j=1,\cdots , q$. Many dictionary learning methods are proposed in literature^42,43^. One can learn the dictionary $D_{i}$ from extracted patches $X_{i}$ by the following formula:

8 $$\begin{aligned} \mathop {\min }\limits _{D_i, \{\alpha _{ij}\}_{j=1}^q}\sum \limits _{j=1}^q\left\{ \frac{1}{2}\left\| {{x}_{ij}}-D_i{{\alpha }_{ij}} \right\| _{2}^{2}+\lambda {\left\| {{\alpha }_{ij}} \right\| }_{1}\right\} \end{aligned}$$

where $$ D_i\in R^{p \times n}$$ is the learned dictionary, $$\alpha _{ij}\in R^n$$ is the representation coefficiences of $$x_{ij}$$ under $$ D_i$$, and $$\lambda $$ is a hyper-parameter. Once the dictionary $$D_{i}$$ is known, the representation coefficient $$\alpha _{i}$$ for the current patch $$x_i$$ can be obtained by solving the optimization problem of formula (7). The most common method for solving formula (7) is the LARS-lasso algorithm^44,45^.

Suppose $\alpha _i^*$ is the optimum solution of formula (7), the background $x_{iB}$ of the current patch $x_i$ is estimated by $D_i\alpha _i^*$ the linear combination with the nonzero elements of $\alpha _i^*$ and the corresponding atoms in $D_i$. When $x_{iB}$ is reconstructed under $D_i$ coming from $X_{i}$ through adaptively choosing the atoms, the local illumination variations between $x_{i}$ and $y_{i}$ is adaptively corrected by adjusting the representation coefficient of the dictionary at the same time.

The change regions appearing in the current patch $x_i$ don’t show in the reference patch $y_i$ and its neighborhood $X_{i}$. Hence the changes aren’t encoded in the dictionary $D_{i}$. These variation features are not projected into the subspace formed by $X_{i}$ but assigned to the error component portion of $x_{i}$ and $x_{iB}$.

**Figure 13:**
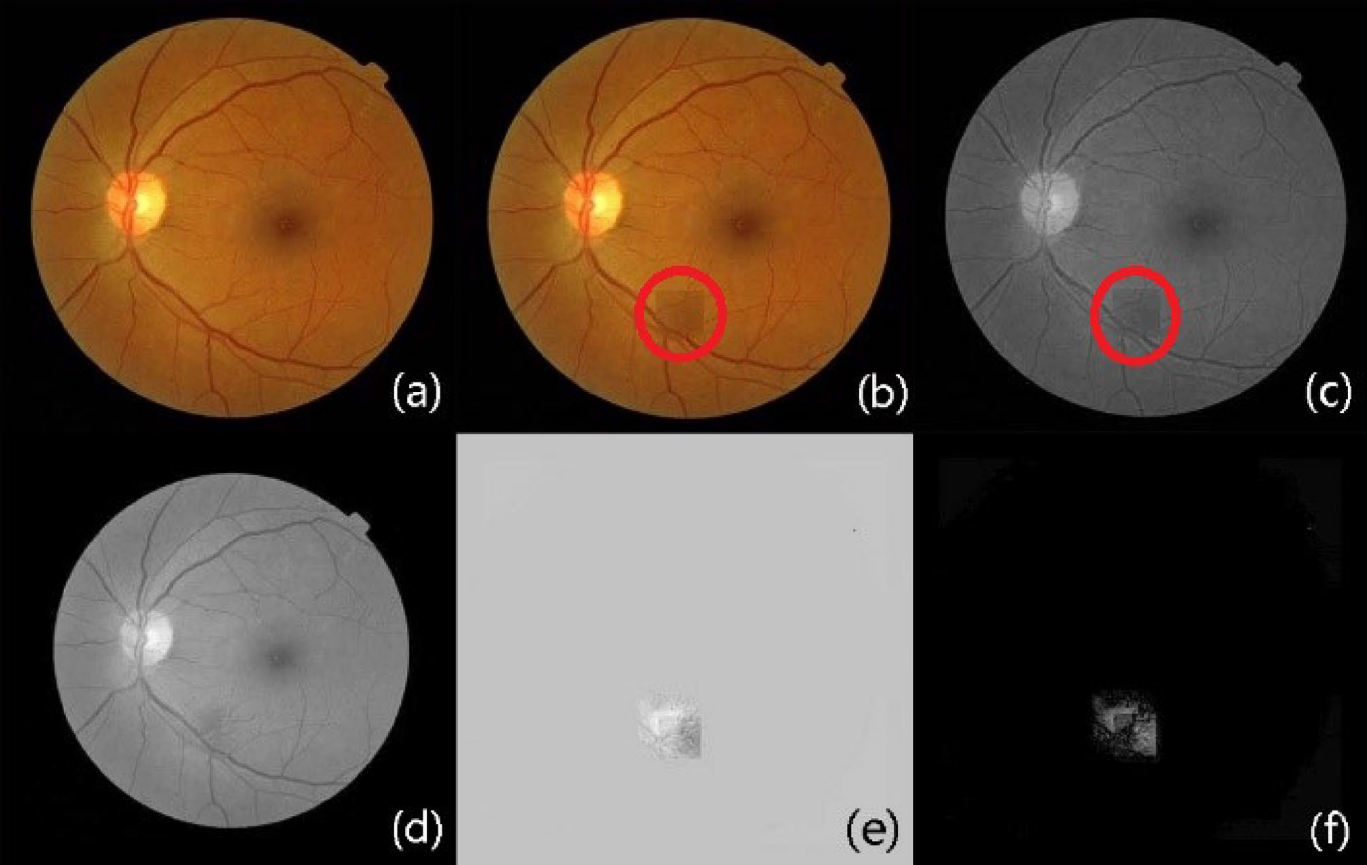
The result of simulated image pair. (**a**) The normal retinal fundus image from DRIVE; (**b**) the image attached with marked by the red circle; (**c**) grayscale image of (**b**); (**d**) the reconstructed background of (**c**) based on the proposed method; (**e**) the difference image of (**c**) and (**d**); (**f**) the absolute difference image of (**c**) and (**d**).

Figure 13 illustrates the proposed algorithms in this paper. The reference image Fig. 13a comes from a normal image from the DRIVE database. The current image Fig. 13b is obtained by affixing a small patch marked with red circle size of $41 \times 41$ in Fig. 13a. The small patch are attached and interpolated linearly with the background by a coefficient of 0.5. Hence Fig. 13a,b constitute a simulated image pair. Figure 13d is the background of Fig. 13c reconstructed by the method proposed in this paper, and Fig. 13e is the result of background subtraction between Fig. 13c,d,f is the detected change region. SRC change detection method proposed in this paper can reconstruct clearly the background of the current image and detect the clean change region.

### Algorithm

The proposed method is roughly divided into three section: preprocessing, estimating the background of the current image and generating change regions. For the given image pair, in the preprocessing step, the images are registered and their illuminations are corrected and normalized to an as possible as uniform intensity level, and the image pairs are grayed out. At the background model estimation step, the background of the current image is reconstructed through a sliding window and sparse representation based on the patch, and the local dictionary is extracted from the neighborhood of the corresponding reference patches. By this mean, the background of the current patch is estimated by sparse representation in the dictionary. Finally, the difference image is produced by subtracting the current image from the reconstructed background, and the change region is generated. The detailed procedure is presented in Table 2.

In order to detect the change regions more efficiently, we give up the dictionary learning processing and take the extracted patches as the atoms of the dictionary instead.

**Table 2:** SRC change detection algorithm proposed in this paper.

**Input:** A pair of registered images: the reference image $I_{1}$ and the current image $I_{2}$	
**Output:** Change regions	
**Step 1**: Gray the image pairs $I_1$ and $I_1$;	
**Step 2:** Pad the outside of the edge in $I_1$ with zeroes;	
**Step 3:** Take the local patch $x_i$ with size $s\times s$ centered on the point *P* from $I_2$, and vectorize $x_i$;	
**Step 4:** Take *N* patches $\{{{x}_{i1}},{{x}_{i2}},\ldots , {{x}_{iq}}\}$ of size $s\times s$ from $I_1$ in a square region around $l\times l$ centered on the pixel $P'$; vectorize N patches and cascade them into a matrix $D_i$, $l>s$;	
**Step 5:** Put $x_i$ and $D_i$ into formula (7) to substitute *x* and *D*, and solve the coefficient $\alpha$ by the LARS-lasso algorithm to obtain the optimum $\alpha ^*$;	
**Step 6:** Estimate the background $x_{iB}$ of $x_i$, and denote $x_{iB}=D\alpha ^*$;	
**Step 7:** Obtain the change regions by calculating $x_i-D\alpha ^*$;	

### Locating big change regions

The patch-based SRC method reconstructs the background well when the change region is small in the current patch with small lesions for most part of the region is consistent with the reference patch and its neighborhood patches, which can be represented by main atoms of the learned dictionary from the reference patch and its neighborhood patches. However, for the big change region, the change covers the most part of the current patch, and the little part left has the similarity with the reference patch and its neighborhood patches. The principle atoms in the learned dictionary can’t be used to represent the current patch, otherwise it will bring big reconstruction error. Hence the subordinate atoms obviously different in feature from the principle atoms are taken to reconstruct the current patch. Such kind of subordinate atoms consist of the subtle information. All the chosen subtle information are combined linearly to represent the current patch which will greatly reduce the reconstruction error. Hence the current patch can’t be reconstructed from the principal components indicating the background in the dictionary.

For the big change region, the current patch is only represented by the less important components in the dictionary, which means that the change region is projected to the subspace consisting with the reconstruction error. The current patch is reconstructed by the less important atoms, and the subtraction between the current patch and the reconstructed patch still shows small value. In this case, background subtraction doesn’t indicate the change region correctly.

However, the SRC-based method provides an approximate estimation about the border of the big change regions. In general, the current patch including the boundary of change region shows the big similarity to the primary atoms in the dictionary. The background of the current patch is reconstructed correctly by sparse representation, and the change is obtained from the reconstruction error. Hence the patch-based SRC method can detect the outline of the big change region as a coarse segmentation for the change region. The SRC method is robust to the local illumination, thus it is combined with the other change detection method to obtain a better performance. IRHSF is specially designed for the fundus images, and the image difference with IRHSF provides a good estimation for change regions. SRC combined with the IRHSF-based image difference improves the detection results and removes the distraction of the local intensity.
